# Local Diagnostic Reference Levels for Pediatric Interventional Cardiology Procedures in Argentina

**DOI:** 10.3390/children10121877

**Published:** 2023-11-30

**Authors:** Patricia Azcurra, Fernando Leyton, Victorio Lucini, Marcelo Rivarola, Luis Trentacoste, Adela Marques, Juan Chiabrando, Ignacio Seropian, Nicolas Mundo, Carlos Ubeda, Carla Agatiello

**Affiliations:** 1 Interventional Cardiology Department, Hospital Italiano de Buenos Aires, Buenos Aires C1199ABB, Argentina; 2 Personal Dosimetry Laboratory (LABODOP), School of Medical Technology, Faculty of Health Sciences, University of Tarapacá, Arica 1010072, Chile

**Keywords:** interventional cardiology, pediatrics, local diagnostic reference levels, kerma area product, radiation dose

## Abstract

The aim of this work was to propose a preliminary local diagnostic reference levels (DRL) for pediatric interventional cardiology (PIC) procedures in Argentina, for different ranges of age and weight. This work has been conducted in the framework of the “Optimization of Protection in Pediatric Interventional Radiology in Latin America and the Caribbean” (OPRIPALC) program coordinated by the World Health Organization and the Pan American Health Organization in cooperation with the International Atomic Energy Agency to ensuring that radiation exposures of pediatric patients are the minimum necessary during fluoroscopy-guided interventional procedures. The local DRL values presented in this paper by weight group and age group were 7.1 Gy·cm^2^ (<5 kg), 10.7 Gy·cm^2^ (5–15 kg), 18.0 Gy·cm^2^ (15–30 kg), 15.9 Gy·cm^2^ (30–50 kg), and 28.2 Gy·cm^2^ (50–80 kg) and 5.3 Gy·cm^2^ (<1), 11.2 Gy·cm^2^ (1 to 5<), 19.6 Gy·cm^2^ (5 to 10<), and 21.4 Gy·cm^2^ (10 to 16<), respectively. Our dose results are among the values found in other international studies; however, there is great potential for dose optimization.

## 1. Introduction

One of the main causes of irradiation of the population due to artificial sources is medical practice. Procedures in interventional cardiology can produce high doses of radiation in patients. In this sense, children and pediatric patients are more sensitive to the adverse effects of radiation compared to adults [1,2,3,4]. Therefore, attitudes and measures are necessary to control and optimize radiation doses for the population.

The constant advancement of medical technology has allowed for the addition of treatments to pathologies as complex as those addressed PIC facilities, improving the quality and life expectancy of the pediatric population suffering from congenital pathologies, thus increasing their frequency compared to surgeries to treat these pathologies [5]. In order to offer these benefits in the catheterization facilities, angiographic systems that emit ionizing radiation are used. The uncontrolled use of this radiation and the complexity of the interventional procedures, added to the greater sensitivity of radiation in children and their longer life expectancies, have a greater possibility of generating long-term stochastic effects such as the incidence of cancer [4,5,6,7,8].

This work has been conducted in the framework of the “Optimization of Protection in Pediatric Interventional Radiology in Latin America and the Caribbean” (OPRIPALC) program coordinated by the World Health Organization and the Pan American Health Organization in cooperation with the International Atomic Energy Agency [9,10].

One of the objectives of the OPRIPALC project has been to define optimization strategies based on a collection of patient doses along with image quality assessment, from a sample of representative hospitals in different Latin American and Caribbean countries proposing and using local, national, or regional diagnostic reference levels (DRLs) [11].

The International Commission on Radiological Protection (ICRP) recommends the use of DRLs as an effective tool for dose optimization and radiological protection of patients undergoing diagnostic or interventional procedures [12]. DRLs were developed from the concept of investigation or action levels or reference levels defined in ICRP 60 [13]. These levels provide a useful way of structuring radiation protection procedures in the field of occupational and public exposure. In the year 1996, both the International Atomic Energy Agency (IAEA) with publication Basic Safety Standard [14] and the ICRP in publication 73 [15] definitely recommend the use of DRLs in medical exposure. The DRLs will be used as a test to identify situations where doses are unusually high. If the reference level is constantly exceeded, procedures and equipment must be reviewed in order to determine if radiation protection has been duly optimized; otherwise, if the levels are not exceeded, measures must be taken to reduce the dose. On the other hand, if the doses are well below DRLs, there should be a review of the image quality obtained.

The ICRP 73 stated that “reference levels for therapeutic procedures are not appropriate”. However, after discussing and analyzing various studies on the subject, the ICRP in 2001 state that “for fluoroscopically guided interventional procedures, DRLs, in principle, could be used to promote the management of patient doses with regard to avoiding unnecessary stochastic radiation risks” [16]. Then, in 2009, with the publication of the IAEA safety reports series No. 59 [17], it states that it is possible to establish guidance levels for complex diagnostic and therapeutic procedures as long as the complexity of the procedure is taken into account. It further states that guidance levels do not apply to individual patients and should allow for higher exposures if duly justified.

Currently in Argentina and the region, there are few studies on diagnostic reference levels in PIC procedures. In the region, there are studies carried out in Brazil [18], Chile [19,20], Costa Rica [21], and those carried out within the framework of the OPRIPALC [22] program. In the study by Ubeda et al. [22], three centers from Argentina participated, which contributed with procedures, but DRLs were not defined for the country.

The aim of this work was to propose preliminary local DRLs for PIC procedures in Argentina, for different ranges of age and weight.

## 2. Materials and Methods

All PIC procedures performed at the Hospital Italiano of Buenos Aires from May 2022 to June 2023 were recorded. For each patient, the procedure identification, age, gender, weight, height, kerma area product (*P*_ka_) [12,23], cumulative air kerma at patient entrance reference point (*K*_a,r_) [12,24], and fluoroscopy time (FT) were registered. The Digital Imaging and Communication in Medicine—radiation dose structured report (DICOM-RDSR) was unavailable for our system; for data recording, retrospective manual data collection was performed from the procedure dose summaries issued by each angiography system stored in the Picture Archiving and Communication System.

The X-ray angiography systems (XRA) Siemens Artis Zee (manufactured in 2008), a Philips Allura FD10 (manufactured in 2010) and a Philips Allura Clarity FD20 (manufactured in 2015) were used. All XRA have the fluoroscopy save tool that was used under the discretion of the team performing the case. The protocols used were the pediatric programs of each team selected for each particular patient. Pediatric protocols available on Allura equipment are: pediatric cardiac <40 kg, >40 kg with cine acquisitions; 15 image per second (IPS) low, 25 IPS normal, single shot exposure, 15 IPS normal, and 25 IPS normal. Fluoroscopy available: low–normal–extra. Available weight range: <5 kg, 5–15 kg, 15–40 kg, 40–55 kg, 55–70 kg, 70–90 kg, and >90 kg. Pediatric protocols available on Artis Zee equipment are: Card Ped < 20 kg; Card Ped < 40 kg; Card Ped < 12 kg; Card Ped 12–40 kg; Card Low Dose. Fluoroscopy available: low, medium, and high (FL−, FL, FL+).

The three XRA are subject to a quality control program in accordance with Argentine law and the IAEA-TECDOC-1958 document [25]. The verification tests of the *K*_a,r_, the calibration of the dose of the XRA, and the image quality control, among other tests, were carried out. The institution has a Radiological Safety Committee, which keeps the quality control program updated, in addition to the Interventional Cardiology service, which has a professional responsible for the radiation protection of the patient and occupationally exposed individuals. The hospital is accredited by the Joint Commission International and the catheterization laboratories are also accredited by the Argentine College of Interventional Cardiologists.

The quantities and symbols recommended by the International Commission on Radiation Units and Measurements and the ICRP to establish DRL were used. The three XRA have a parallel plate ionization chamber located in the head of the X-ray tube to measure the values of the *P*_ka_ or also called the dose-area product (DAP) and the *K*_a,r_ or cumulative dose at the patient entrance reference point (CD). Total *P*_ka_ and *K*_a,r_ for each procedure were not corrected by the correction factor derived from the attenuation of the table and mattress; specific to the X-ray beam qualities used in the XRA [22].

The methodology to collect patient dose data and to calculate DRLs was used by the OPRIPALC project [11,26]. To establish the local DRLs, the procedures were grouped into diagnostic, therapeutic, and all together (diagnostic + therapeutic), then subgroups were made for 4 age ranges (<1 year; 1 to <5 years; 5 to <10 years; and 10 to <16 years) and 5 weight groups (<5 kg; 5–15 kg; 15–30 kg; 30–50 kg; and 50–80 kg). The mean, the median, and the 75th percentile of the *P*_ka_ value, the *K*_a,r_, and the FT were obtained. According to ICRP 135 document [12], the 75th percentile of the *P*_ka_ was used to establish the DRL for each group. 

Statistical analysis was performed with the Stat graphic plus 5.0 software was used [27]. The Mann–Whitney test (95% confidence level) was used to compare median values for the *K*_a,r_ between the procedure groups.

The study was approved by the local Institutional Review Board (No. 6407) and was registered at the Computerized Registry of Health Research Platform of Buenos Aires (No. 6534).

## 3. Results

Data from 222 consecutive procedures were evaluated in the study period, including 101 female and 121 male pediatric patients. Patients underwent 51 diagnostic procedures and 171 therapeutic procedures. A total of 154 PIC procedures (40 diagnostic, 114 therapeutic) were performed in the Artis Ze catheterization laboratory, 61 PIC procedures (11 diagnostic, 50 therapeutic) were performed in the Phillips Allura FD10 catheterization laboratory, and 7 PIC procedures (7 therapeutic) were performed in the Phillips Allura FD20 catheterization laboratory.

Table 1 and Table 2 shows of results of the demographic characteristics (frequency, height, and weight) of the patients by age and weight groups, respectively.

Preliminary proposed local DRLs are presented at the 75th percentile of the *P*_ka_ in Table 3, Table 4 and Table 5 by procedures, age groups, and weight groups, respectively. Statistically significant differences were found between the diagnostic and therapeutic procedures for *P*_ka_ (Table 3). We used a Mann–Whitney (Wilcoxon) W test to compare medians at the 95.0% confidence level, *p*-value = 0.0192.

Table 3 summarizes the median, mean, standard deviation, and 75th percentile values for *P*_ka_, *K*_a,r_, and FT for all the collected pediatric procedures (diagnostic + therapeutic) and frequencies by type procedures. The Q75 of the *P*_ka_ corresponds to the preliminary proposal of the local DRLs by type of procedure.

For the general cohort (All procedures, Table 3), the median *P_ka_*, *K_a,r_*, and TF were 7.3 Gy·cm^2^, 89 mGy, and 12.5 min, respectively. The 75th percentile values for all procedures were 15 Gy·cm^2^, 175.8 mGy, and 17.5 min, respectively.

Table 4 and Table 5 summarize the sample size, median, mean, and 75th percentile values for *P*_ka_, *K*_a,r_, and FT for all the collected pediatric procedures (diagnostic + therapeutic) by age and weight group. The Q75 of the *P*_ka_ corresponds to the preliminary proposal of the local DRLs by age and weight group.

Comparison of median *P*_ka_ values for PIC procedures by weight and age group reported in this and other studies are shown in Table 6 and Table 7 (values adapted from Ubeda et al. [22]). Note that the comparison was made for median values because the DRLs (75th percentile) were not reported in all the studies.

In Table 8, the procedures that reached the highest values of *P*_ka_, *K*_a,r_, and FT were recorded and identified.

## 4. Discussion

This study represents a first dose measurement experience to estimate DRLs in patients undergoing PIC in Argentina. 

Our study includes 222 cases, which is relatively lower than those reported by Glazt et al. [33], with a sample size of 2265 cases, and also an Artis Zee catheterization system was used. The values of the median and (75th percentile) were recorded at 7.6 (28.1) Gy·cm^2^, 135 (433) mGy, and 15 (27) min. Although the *P*_ka_ is similar in both studies, it was observed that the *K*_a,r_ and FT can be very different. This difference in values may be due to the fact that patients over 80 kg were included in the Glazt et al. study. This informal comparison between centers, for the values of *P*_ka_, *K*_a,r_, and FT reinforces the idea that any optimization process must consider at least these three variables. The study by Glazt et al. was not included in Table 6 and Table 7 because another weight and age group were used.

Our results are relatively similar to those reported by Manica et al. [18], developed in Brazil, with a number of 429 procedures. In that study, the median and 75th percentile of the dose-area product (equivalent to *P*_ka_) and fluoroscopy time were 7.4 and 17.9 Gy·cm^2^ and 9 and 15 min, respectively. Although our *P*_ka_ values were slightly lower, our FT values were higher by at least 2.5 min than those registered by Manica et al. The study by Manica et al. used another stratification by age group and did not use stratification by weight group. Manica et al. states that only for patients older than 15 years, statistically significant differences were found between the *P*_ka_ for diagnostic and therapeutic procedures, this result could be related to the fact that a small number of patients was used. In the study, the quotient between *P*_ka_ and the weight for the different types of procedures was calculated. No statistically significant differences were observed in the *P*_ka_/weight ratio between the diagnostic and therapeutic procedures. Manica et al. state that there was a statistically significant difference between the subgroups of intervention procedures and the DAP/weight ratio. This interesting approach should be considered in future studies to see the possibility of DRLs by type of procedure.

The registry of the procedures that reach the highest values of *P*_ka_, *K*_a,r_, and TF (Table 8) could potentially identify the most complex procedures that use the highest values of dose and exposure time. These extreme values can also help to improve clinical practice and the radiological protection of the patient and occupationally exposed staff. These results are lower compared to the higher values of *P*_ka_ reported by Ubeda et al. [22] conducted in 18 centers that participate in the OPRIPALC program. The *K*_a,r_ value is below the threshold value for deterministic effects of 2000 mGy (e.g., transient erythema [30]). Considering the dose levels of this study, together with the risk management strategies of the organization and with permanent training in radiation protection that promotes a culture of safety in the clinical team, the probability of skin damage is negligible.

The ICRP states that the process to set and update DRLs should be flexible for procedures where few data are available such as PIC procedures or where data are available from only one or a few centers. It is necessary to allow initial DRLs to be derived from these data while a larger survey is conducted [12]. 

In Table 4, the values of *P*_ka_, *K*_a,r_, and TF by age group are observed; the *P*_ka_ and the *K*_a,r_ increase as the age group increases, this same trend occurs in all the studies presented in Table 7, except for the study by Ishibashi et al. The TF does not show a clear trend; however, the 75th percentile of the TF decreases when the age group increases.

In Table 5, the results of *P*_ka_, *K*_a,r_, and TF are presented for the groups separated by weight. The *P*_ka_ and *K*_a,r_ have a tendency to increase as the weight group increases; however, for the case of weight between 30 and 50 kg, the *P*_ka_ and *K*_a,r_ decrease. The TF was higher for the 5 to 15 kg group and lower for the 30 to 50 kg group. Consistent with the studies by Verghese et al. [30] and Glatz et al. [33], which have a large volume of cases, *P*_ka_, *K*_a,r_, and TF increased with increasing age and weight. In Rassow’s study [34], the median *P*_ka_ also increases as the age group increases.

A database with 222 patients is a limitation of our study, and this could explain the break in the trend for *P*_ka_ and *K*_a,r_ described in Table 5 with small n values for certain weight groups. This lack of trend in the increase in *P*_ka_ and *K*_a,r_ as the age group increases is observed for therapeutic protocols in the study by Ishibashi et al. [32] with a sample of 333 patients. Then, in order to overcome this limitation, obtaining a larger database in pediatric interventional cardiology is posed as a task in the medium or long term, in the sense that the study by Rassow et al. [34] had a registry of 2114 patients who underwent cardiac catheterization and cineangiocardiography between 22 April 1984 and 8 July 1996. The study states that the number of patients collected in 12 years of study was 294 patients for the age range between 10 and 15 years. On the other hand, a multicenter study conducted by Ubeda et al. [22] declared that totals of 728 and 685 procedures were collected from December 2020 to December 2021 by age and weight groups, respectively.

In the communication by Natali et al. [35], the dose was studied in 1410 pediatric interventional cardiology procedures between the years 2016 and 2019. This retrospective study focused on the analysis of the different approaches for the placement of central venous catheters in pediatric patients to prevent errors in selection. The study states that “the *P*_ka_ and effective dose values were significantly reduced in 2018–2019 compared to 2016–2017”. In 2019, a mean *P*_ka_ value of 0.031 Gy·cm^2^ was obtained for all procedures. The registration of procedures and their doses is a challenge in the region to keep the database active for years and generate databases with large volumes of procedures to implement optimization procedures and to study the behavior of different variables that affect the dose to patients, such as the complexity of the procedures, operator experience, among others.

Table 6 and Table 7 show the median *P*_ka_ values by age group and weight group for all the PIC procedures reported in this article and other similar studies. In both Table 6 and Table 7, there is a great dispersion of *P*_ka_ values, and the range of values goes from 0.6 to 23.6 Gy·cm^2^ and 0.9 to 24.7 Gy·cm^2^ by weight group and age group, respectively. Our results are within these ranges. In the case of the values by weight group (Table 6), between 5 and 30 kg, there is a great potential for optimization since our results are higher compared to the other studies by factors of 1.9, 1.8, and 1.6 compared to the groups <5 kg, 5 to 15<, and 15 to 30<, respectively. These results show a wide margin to optimize procedures, such as in the performance of the equipment (characterization), the settings used, and the examination protocols, which should be customized to suit the patient’s weight as corrective actions to reduce the patient dose.

In Table 7, our *P*_ka_ values were the third highest for the groups between 1 and 10 years. In the case of the group between 10 to <16 years, our median value was the fourth lowest value among the 9 studies presented. In addition, it is observed that the *P*_ka_ increases as age increases, confirming this behavior in all the studies in Table 7. As in Table 6, the potential for optimization by age group is confirmed.

Some limitations identified during the study are summarized in this paragraph. The sample used to obtain these preliminary results was not very large, especially when referring to the different age and weight groups. Reported radiation dose was manually obtained by reviewing each case and may have introduced some mistakes. In this sense, automated software that prospectively makes collections and storage radiation dose structure reports will facilitate further analyses. Analysis that related dose level to procedure complexity and operator experience was not performed, and this should be considered in the next studies.

## 5. Conclusions

Within the framework of the OPRIPALC project, this study presents the first preliminary values of local diagnostic reference levels in Argentina in pediatric interventional cardiology for diagnostic and therapeutic procedures by age and weight group. To the best of our knowledge, this study has the largest database of doses to patients undergoing pediatric interventional cardiology procedures in the country and is the third center to join this initiative in Argentina [22].

The local DRLs values reported in this study were 9.7 and 17.5 Gy·cm^2^ in pediatric patients undergoing diagnostic and therapeutic interventional cardiology procedures, respectively. The local DRLs values presented in this paper by weight group and age group were 7.1 Gy·cm^2^ (<5 kg), 10.7 Gy·cm^2^ (5–15 kg), 18.0 Gy·cm^2^ (15–30 kg), 15.9 Gy·cm^2^ (30–50 kg), and 30.5 Gy·cm^2^ (50–80 kg) and 5.3 Gy·cm^2^ (<1), 11.2 Gy·cm^2^ (1 to 5<), 19.6 Gy·cm^2^ (5 to 10<), and 21.8 Gy·cm^2^ (10 to 16<), respectively. Our dose results are among the values found in other international studies; however, there is great potential for dose optimization because there are centers that manage to perform diagnostic and therapeutic procedures with 50% less dose. Therefore, it is urgent to continue with the investigations to increase the number of cases and consider factors such as the complexity of the procedure among other variables.

The local DRL in this study represents the first step in the optimization processes, allowing us to strengthen the security culture together with the continuous improvement of interventional procedures, guaranteeing that pediatric patients receive as low doses as reasonably possible, promoting excellence and solidarity in the whole-human team associated with cardiological intervention.

## Figures and Tables

**Table 1:** Demographic characteristics for the sample of patients, number of patients (n), mean, maximum (max), minimum (min) values of height, and weight by age group.

Age Group (Years)	n	Height (m)			Weight (kg)		
		Mean	Max	Min	Mean	Max	Min
<1	28	0.58	0.70	0.45	5.2	8.0	2.5
1 to <5	87	0.86	1.23	0.47	12.3	24.0	3.2
5 to <10	68	1.08	1.44	0.68	20.3	52.5	10.2
10 to <16	39	1.43	1.83	1.21	41.1	66.5	21.8

**Table 2:** Demographic characteristics for the sample of patients, number of patients (n), mean, maximum (max), minimum (min) values of height, and weight by weight group.

Weight Group (kg)	n	Height (m)			Weight (kg)		
		Mean	Max	Min	Mean	Max	Min
<5	21	0.5	0.6	0.5	3.9	4.9	2.5
5 to <15	80	0.8	1.1	0.6	10.3	14.8	5.2
15 to <30	85	1.1	1.4	0.7	20.0	29.8	15.0
30 to <50	22	1.4	1.6	1.2	36.6	49.8	30.0
50 to <80	14	1.6	1.8	1.4	56.7	66.5	50.7

**Table 3:** Statistical values for *P_ka_*, *K_a,r_*, and FT by type of procedure. Sample size (n), standard deviation (SD), and 75th percentile (Q75).

		*P*_ka_ (Gy·cm^2^) *				*K*_a,r_ (mGy)				FT (min)			
Procedures	n	Median	Mean	SD	Q75	Median	Mean	SD	Q75	Median	Mean	SD	Q75
Diagnostic	51	5.7	7.2	5.9	9.7	45.0	68.3	60.8	90.5	7.4	8.6	4.5	11.8
Therapeutic	171	8.5	13.5	17.1	17.5	101.0	140.5	122.5	193.5	13.6	16.4	11.3	20.3
All	222	7.2	12.1	15.5	15.3	88.5	123.9	115.3	178.1	12.5	14.6	10.7	17.5

* Statistically significant differences were found between the diagnostic and therapeutic procedures for *P*_ka_ (*p* < 0.019).

**Table 4:** For all procedures, statistical values for *Pka*, *Ka,r*, and FT by age group. Sample size (n) and 75th percentile (Q75).

Age Group		*P*_ka_ (Gy·cm^2^)			*K*_a,r_ (mGy)			FT (min)		
Years	n	Mean	Median	Q75	Mean	Median	Q75	Mean	Median	Q75
<1	28	5.1	3.7	5.3	99.0	73.2	124.5	15.3	12.7	18.6
1 to <5	87	10.0	5.3	11.2	105.6	75.0	149.5	14.1	12.5	18.1
5 to <10	68	13.6	10.9	19.6	129.4	113.0	187.0	16.3	13.2	17.7
10 to <16	39	18.8	12.8	21.8	172.9	97.0	219.5	12.5	8.6	16.5

**Table 5:** For all procedures, statistical values for *P*_ka,_ *K*_a,r_, and FT by weight group. Sample size (n) and 75th percentile (Q75).

Weight Group		*P*_ka_ (Gy·cm^2^)			*K*_a,r_ (mGy)			FT (min)		
kg	n	Mean	Median	Q75	Mean	Median	Q75	Mean	Median	Q75
<5	21	4.9	3.3	7.1	95.8	70.0	120.0	15.5	12.3	20.4
5 to <15	80	9.5	5.3	10.7	109.2	78.5	141.3	14.6	13.3	18.1
15 to <30	85	13.8	10.9	18.0	134.8	107.0	189.7	15.8	12.5	19.6
30 to <50	22	10.9	8.5	15.9	131.5	73.5	148.8	12.1	8.5	16.0
50 to <80	14	28.5	18.8	30.5	171.9	136.5	213.5	10.7	9.9	14.2

**Table 6:** Comparison of median *P_ka_* values for PCI procedures by weight group reported in this and other studies (values adapted from Ubeda et al. [23]).

Weight Group (kg)	Buytaert et al. [28] (Gy·cm^2^)	Ubeda et al. [20] (Gy·cm^2^)	Ubeda et al. [22] (Gy·cm^2^)	This Paper (2023) (Gy·cm^2^)
<5	0.6	1.9	1.8	3.7
5 to <15	0.7	2.7	2.5	5.3
15 to <30	1.9	6.9	3.3	10.9
30 to <50	7.1	14.2	6.2	8.5
50 to <80	8.7	12.6	23.6	18.8

**Table 7:** Comparison of median *P_ka_* values for PCI procedures by age group reported in this and other studies (values adapted from Ubeda et al. [22]).

Age Group (Years)	Martinez et al. [29] (Gy·cm^2^)	Verghese et al. [30] (Gy·cm^2^)	Ubeda et al. [19] (Gy·cm^2^)	Ubeda et al. [21] (Gy·cm^2^)	Kottou et al. [31] (Gy·cm^2^)	Ubeda et al. [20] (Gy·cm^2^)	Ubeda et al. [22] (Gy·cm^2^)	Ishibashi et al. [32] (Gy·cm^2^)	This Paper (2023) (Gy·cm^2^)
<1	1.9	4.6	0.9	1.1	2	2.1	1.9	5.2	3.7
1 to <5	2.9	8.3	1.5	1.5	3	4.7	2.6	12.3	5.3
5 to <10	4.5	11.5	2.1	2.7	7	6.3	3.6	7.3	10.9
10 to <16	15.4	24.7	5.0	8.4	14	13.6	11.5	25.5	12.8

**Table 8:** The three procedures that reached the highest values of *P_ka_*, *K_a,r_* and FT.

Procedures	Age (Years)	Weight (kg)	Height (m)	*P*_ka_ (Gy·cm^2^)	*K*_a,r_ (mGy)	FT (min)
Pediatric cardiac catheterization + angioplasty of aortic coarctation + pulmonary artery collateral embolization	10	54	1.41	131.6	147	17.5
Pediatric cardiac catheterization + ductus closure	16	56	1.62	63.4	680	19.3
Pediatric cardiac catheterization + ATC Pulmonar	5	21	0.75	32.9	445	60

## Data Availability

The data presented in this study are available on request from the corresponding author. The data are not publicly available due to ethical and privacy restrictions.
